# SOD1/Rag2 Mice with Low Copy Number of SOD1 Gene as a New Long-Living Immunodeficient Model of ALS

**DOI:** 10.1038/s41598-018-37235-w

**Published:** 2019-01-28

**Authors:** M. Majchrzak, K. Drela, A. Andrzejewska, P. Rogujski, S. Figurska, M. Fiedorowicz, P. Walczak, M. Janowski, B. Lukomska, L. Stanaszek

**Affiliations:** 1 NeuroRepair Department, Mossakowski Medical Research Centre, Polish Academy of Sciences, Warsaw, Poland; 2 Laboratory for Genetically Modified Animals, Mossakowski Medical Research Centre, Polish Academy of Sciences, Warsaw, Poland; 3 Department of Experimental Pharmacology and Small Animal Magnetic Resonance Imaging Laboratory, Mossakowski Medical Research Centre, Polish Academy of Sciences, Warsaw, Poland; 4 Johns Hopkins University School of Medicine, Institute for Cell Engineering, Division of MR Research, The Russell H. Morgan Department of Radiology and Radiological Science, Baltimore, MD, USA; 5 Department of Neurosurgery, School of Medicine, Collegium Medicum, University of Warmia and Mazury, Olsztyn, 10-719 Poland

## Abstract

The most recent research concerning amyotrophic lateral sclerosis (ALS) emphasizes the role of glia in disease development. Thus, one can suspect that the effective therapeutic strategy in treatment of ALS would be replacement of defective glia. One of the basic problems with human glial progenitors (hGRPs) replacement strategies is the time needed for the cells to become fully functional *in vivo*. The lifespan of most popular high copy number SOD1 mutant mice might be too short to acknowledge benefits of transplanted cells. We focused on developing immunodeficient rag2^−^/^−^ model of ALS with lower number of transgene copies and longer lifespan. The obtained hSOD1/rag2 double mutant mice have been characterized. QPCR analysis revealed that copy number of hSOD1 transgene varied in our colony (4–8 copies). The difference in transgene copy number may be translated to significant impact on the lifespan. The death of long- and short-living hSOD1/rag2 mice is preceded by muscular weakness as early as one month before death. Importantly, based on magnetic resonance imaging we identified that mutant mice demonstrated abnormalities within the medullar motor nuclei. To conclude, we developed long-living double mutant hSOD1/rag2 mice, which could be a promising model for testing therapeutic utility of human stem cells.

## Introduction

Amyotrophic lateral sclerosis (ALS) is a progressive neurodegenerative disease affecting upper and lower motor neurons (MNs) in the central nervous system (CNS). Although ALS is not a common illness with an incidence rate of about 1 per 100 000 people a year, the progression of ALS is dramatic as the survival of patients oscillates around 2–5 years from diagnosis^1^. The disease has complex background, the pathomechanism of neurodegeneration is not well understood and the symptoms of the disease differ in time and course. Nonetheless, the majority of patients suffer from muscle weakness (specifically muscles involved in speech, swallowing and limb movement), spasticity and paralysis, which in terminal stage leads to respiratory failure requiring mechanical ventilation support. Until now, no effective cure has been found. The only treatment established so far is limited to alleviate patient’s burden with no effect on the disease progression.

As already mentioned, background of ALS is not yet known neither well described. The results of recent studies incline that several combined mechanisms leading to ALS phenotype are at play rather than one simple mechanism. Among others, the following are taken into consideration: mitochondrial dysfunction, glutamate toxicity, oxidative stress, protein aggregates (e.g. misfolded SOD1), accumulation of neurofilaments, neuroinflammation or failure of glia to support MNs^2,3^.

Amyotrophic lateral sclerosis can be divided into sporadic (sALS) and familial (fALS) form. 5–10% of fALS patients share the autosomal dominant mutations of several genes including: SOD1, ALS2, FUS, ANG, UBQLN2 and C9ORF72^4^. One of the most common rodent models of amyotrophic lateral sclerosis are mice with hSOD1 mutation (Jackson Laboratory; B6SJL-Tg(SOD1*G93A)1Gur/J, also known as SOD1-G93A stock# 002726). Mutation lies in a single amino acid substitution of glycine into alanine at codon 93. Transgenic mice display phenotype resembling ALS, namely hind and forelimb paralysis due to motor neuron degeneration, and shortened lifespan (to approximately 129+/− 9 days).

There is a growing interest in transplantation of stem cells as a therapeutic strategy for ALS. Different approaches are taken into consideration including mesenchymal or neural stem cell treatment in order to support endogenous repair processes or replace defective motor neurons^5^. However recent reports elevated the role of glia in ALS pathology^6,7^ and points the glia replacement as a potential therapeutic treatment^3,8,9^. Irrespectively of cell type and therapeutic mechanism, it is critical to utilize appropriate animal model allowing for conclusive evaluation of a given therapeutic effect. This is particularly true when using donor cells originating from different species (xenotransplantation), the case of studying therapeutic properties of human stem cells in animal models of neurodegenerative diseases. Two important issues have to be considered in studies with xenotransplantation of human cells. First is the problem of immunorejection and second is the time needed for the cells to reveal their therapeutic effect. As it was recently proven by Walczak group, the time needed for human glial progenitors to produce myelin post-surgery is approximately 4 months^10^. The survival time of commonly used mouse ALS model (B6SJL-Tg(SOD1*G93A)1Gur/J) would likely be too short to realize full benefits of stem cell therapy. There are reports indicating that the severity of the disease highly depends on the copy number of mutated SOD1 gene, as well as genetic background^11–13^. In brief, the lower the copy number the less severe the phenotype, and finally the longer the lifespan.

In order to obtain a suitable mouse model for investigation of stem cell therapy in ALS that would also enable us to perform xenotransplantation we decided to cross two mice mutants: (i) B6SJL-Tg(SOD1*G93A)1Gur/J – ALS model with (ii) B6(Cg)-*Rag2*^*tm1.1Cgn*^/J – immunocompromised mice. The B6SJL-Tg(SOD1*G93A)1Gur/J (Jackson Laboratory) is a mouse model commonly used to investigate neuromuscular disorders such as ALS. Aforementioned mice have a transgenic insertion of human mutated SOD1 gene (substitution of glycine into alanine at codon 93) responsible for ALS-like phenotype. The hemizygous mice are fertile, however their lifespan is shortened to around 130 days. Due to the high copy number of the SOD1 gene (10 copies) mice begin to demonstrate paralysis in limbs due to motor neurons loss. The B6(Cg)-*Rag2*^*tm1.1Cgn*^/J, commonly known as rag2 KO mice, have a deletion in exon 3 in *Rag2* gene. This knock-out mutation impairs T and B lymphocytes production, thus affecting immunological response in mutant mice.

The aim of our studies was to create and characterize immunodeficient mouse model of ALS with prolonged lifespan thus enabling longer observation after treatment.

## Results

### Genotyping and phenotypic description

**Figure 1:**
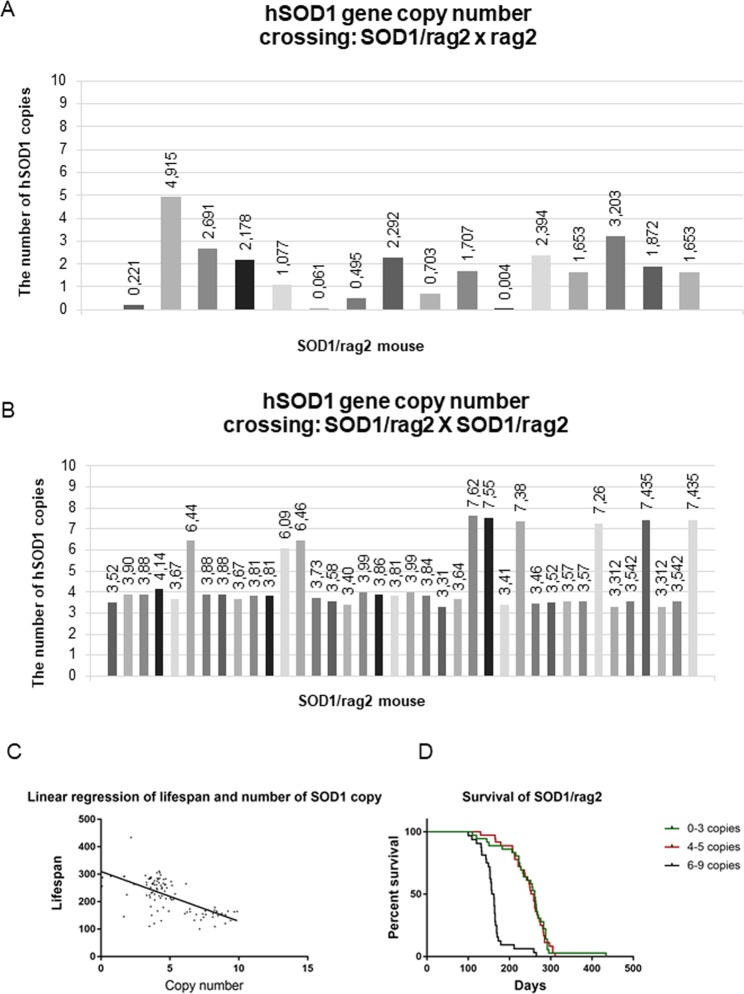
The number of hSOD1 copies in SOD1/rag2 mice colony after crossbreeding: SOD1/rag2 x rag2 (**A**) and SOD1/rag2 x SOD1/rag2 (**B**). The correlation between the copy number of SOD1 transgene and the length of life of SOD1/rag2 mice (**C**). The survival of SOD1/rag2 mice in colony depends on the number of copies of transgene (**D**).

We crossed animals in two ways: (i) m SOD1-G93A/rag2 KO x f rag2 KO (SOD1/rag2 x rag2) and (ii) SOD1-G93A/rag2 KO x SOD1-G93A/rag2 KO (SOD1/rag2 x SOD1/rag2). When mice were crossed in the following pattern: SOD1/rag2 x rag2, the progeny had lower than 3 number of SOD1 copies – ‘very low copy number’ (Fig. 1A). If the second pattern of crossbreeding was applied (SOD1/rag2 x SOD1/rag2) the progeny could be divided into mice with 3–5 copies – ‘low copy number’ and 6–9 copies of hSOD1 gene – ‘high copy number’, respectively (Fig. 1B). In further analysis to facilitate the naming of groups we called last groups 4-copy and 8 copy, respectively. We managed to demonstrate the correlation between the number of SOD1 gene copies and the lifespan of SOD1/rag2 mice (Fig. 1C). Mice lifespan was inversely proportional to the number of transgene copies, with correlation factor r = −0,6563 (p < 0.0001). We wanted to know if we can predict the length of mice lifespan based solely on number of transgene copies. We obtained linear regression line with r^2^ = 0.43, and p < 0.0001 proving that we are able to forecast the average lifespan of mice knowing the number of hSOD1 copies (Fig. 1D.). The group with less than 3 copies of hSOD1 gene lived typically around 265 days (+/− 40 days) similarly to 4-copy number group which on average lived 261 days (+/− 30 days) (Fig. 1C). The 4 copy number mice started to develop hind paw contraction and paralysis 2–3 weeks before death. The group of mice with highest copy number of transgene lived on average around 163 days (+/−13 days) and had their first symptoms visible around 110–120 days after birth, followed by noticeable limb paralysis around the age of 140 days. Due to very low copy number of mutated hSOD1 gene mice with lower than 3 copies of gene were excluded from further analysis and thus further crossbreeding was proceeded based on SOD1/rag2 x SOD1/rag2 pattern.

### MRI analysis of mice brain

**Figure 2:**
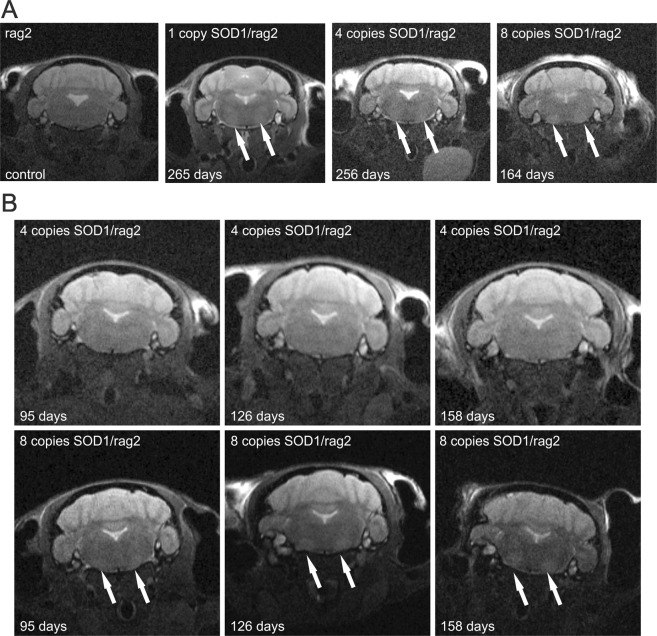
Magnetic resonance imaging of SOD1/rag2 mice. (**A**) MRI of rag2 control mouse and SOD1/rag2 mice in the stage of the disease when the symptoms of the disease are visible (1 copy of SOD1 gene – 265 days, 4 copy of SOD1 gene – 256 days, 8 copies of SOD1 gene – 164 days) showing hyperintensities in T2 weighted images in the area of motor nucleus in medulla. (**B**) Comparison of 4-copy mouse (upper panel) and 8-copy animal (lower panel) at the 95, 126 ad 158 days of living. The hyperintensities in medulla are visible only in the 8-copy animal, pronounced especially during the symptomatic period.

T2-weighted images from magnetic resonance imaging revealed the presence of hyperintensities in the area of facial (VII), parapyramidal and paragigantocellular reticular nuclei in medulla of SOD1/rag2 mice. Changes were visible during the symptomatic period of the disease in animals with 1, 4 and 8 copies of hSOD1 transgene (Fig. 2A). The changes in nuclei were not present in rag2 animals that did not demonstrate any disease symptoms. Moreover, MRI changes were only visible in presymptomatic/symptomatic time of the disease (Fig. 2A,B). The hyperintensities on T2 weighted image were not visible before the onset of the disease in 4-copies mice (Fig. 2B, upper panel), however in highest copy number mice the changes in motor nuclei were already visible at the age of 95 days, while during observation mice did not reveal any visible symptoms such as limb paralysis.

### Immunohistochemical analysis of the spinal cord and mice brain

**Figure 3:**
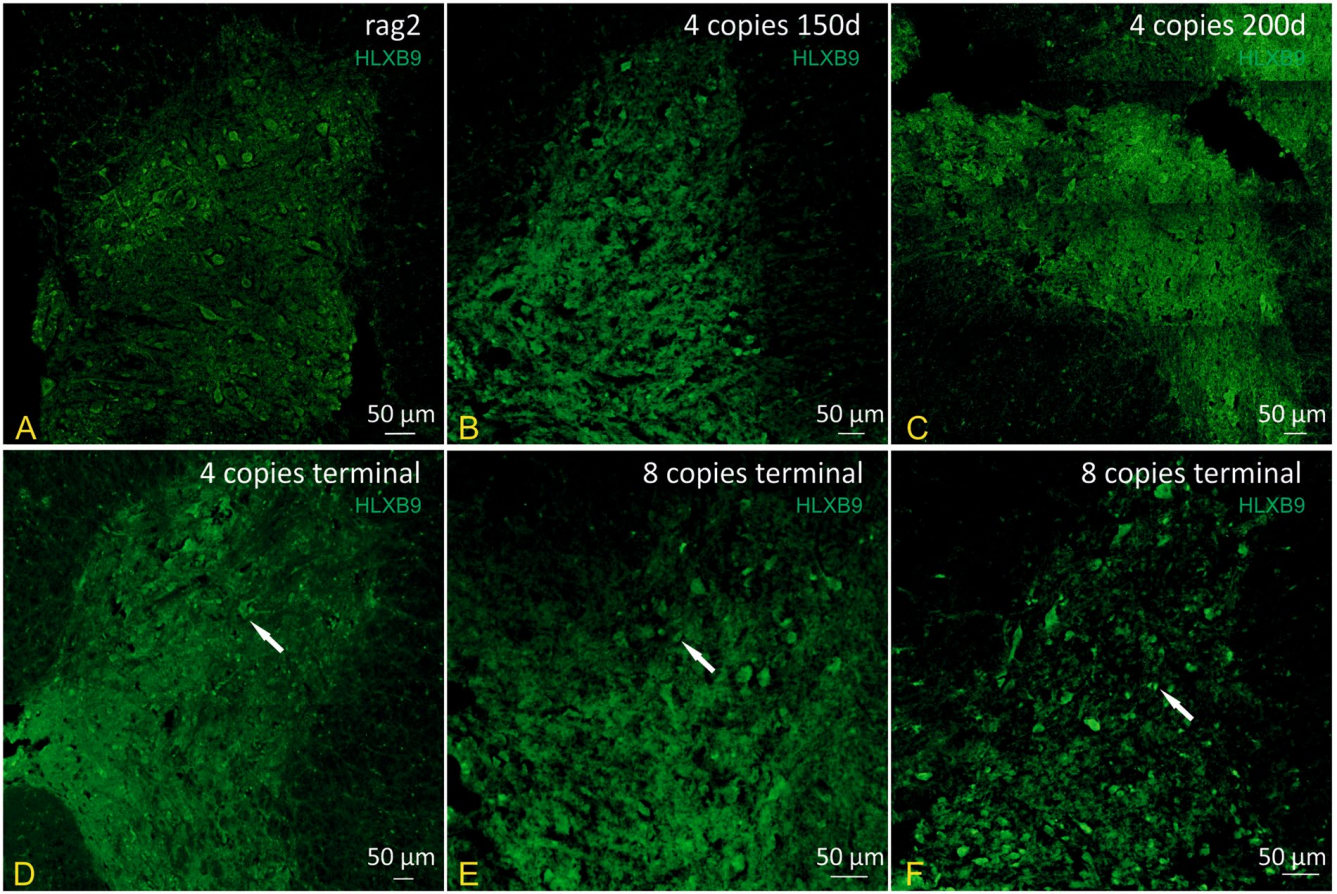
Immunohistochemical analysis illustrating MNs (HLXB9 – green) in spinal cords of 4- and 8- copies SOD1/rag2 mice. (**A**) control rag2 mice with a healthy MNs within spinal cord, (**B**,**C**) 4-copy SOD1/rag2 mice in pre-symptomatic stage (150 and 200 post-natal day, respectively), (**D**) 4-copy SOD1/rag2 mice in terminal stage of the disease, (**E**,**F**) 8-copy SOD1/rag2 mice in terminal stage of the disease.

**Figure 4:**
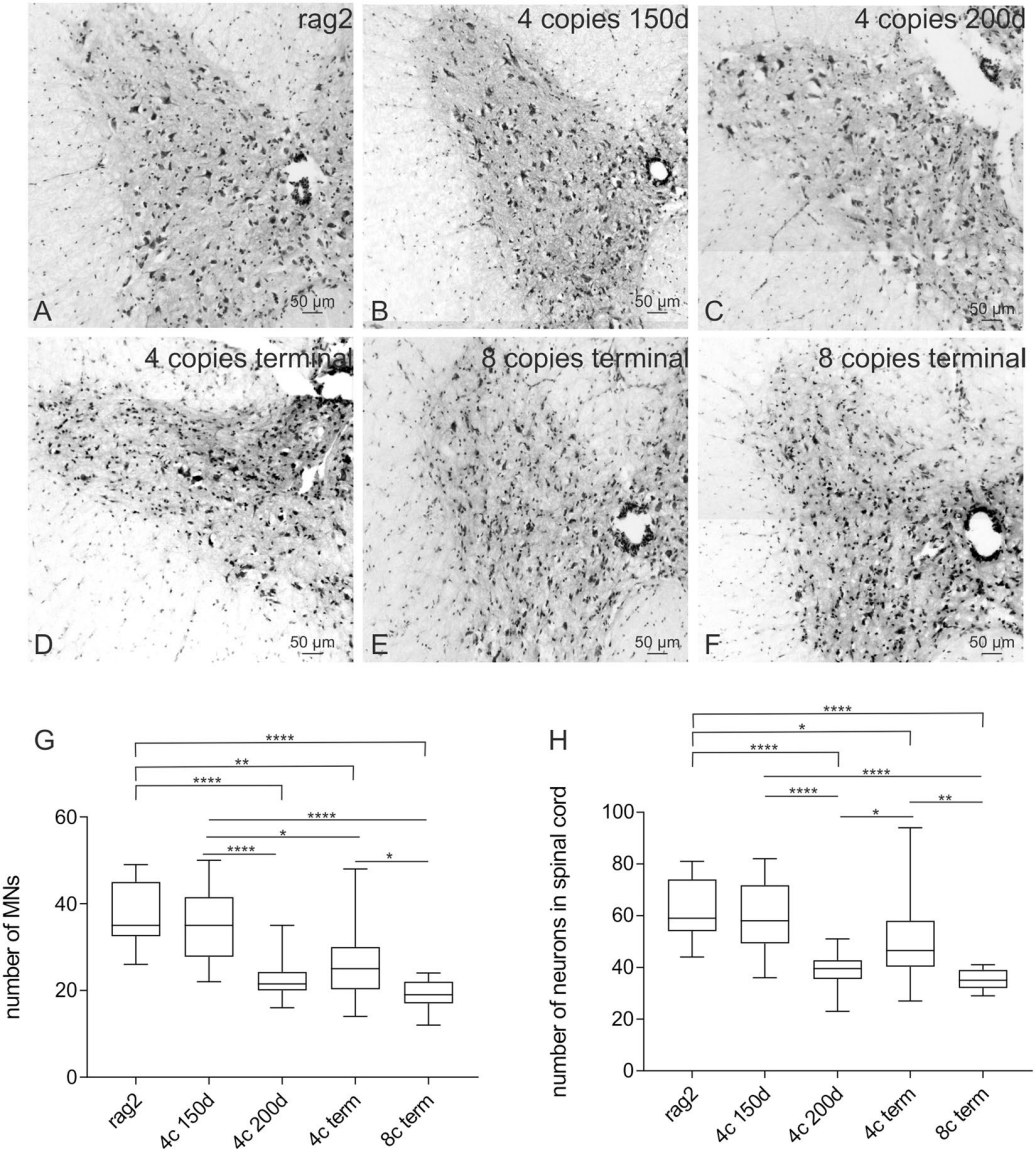
Cresyl violet staining illustrating changes in number of neurons in spinal cords of (**A**) rag2 control animal, (**B**,**C**) 4-copy SOD1/rag2 mice in pre-symptomatic stage (150 and 200 post-natal day, respectively), (**D**) 4-copy SOD1/rag2 mice in terminal stage of the disease, (**E**,**F**) 8-copy SOD1/rag2 mice in terminal stage of the disease. Graphs illustrate number of motor neurons (**G**) and total number of neurons (**H**) counted from sections of spinal cords stained with cresyl violet. *p<0,05; **p<0,01; ****p<0,0001.

The spinal cords of control (rag2) and 4 and 8-copy mice were stained immunohistochemically using anti-HLXB9 antibody in order to visualize and compare motoneurons (MN) in animals that differ in the copy number of hSOD1 gene. Qualitative analysis of all of the animals in terminal stage of the disease, independently of the copy number of SOD1 gene revealed reduced number of MNs (Fig. 3 and 4). The persistent MNs in late symptomatic stage were mostly smaller (Fig. 3D–F, arrows). Some of the 4 copy SOD1/rag2 mice at the age of 200 days had already shown reduced number of MNs (Fig. 3C). At the age of 150d 4 copy number animals have not yet shown any pronounced changes in MNs morphology or number (Fig. 3B). The MNs of 8-copy SOD1/rag2 mice were reduced in number and in size at symptomatic stage of the disease in comparison to rag2 control mice (Fig. 3E,F). Quantitative analysis revealed that the number of MNs in ventral horn of spinal cords of 4-copy number mice decreased significantly at the age of 200 days (p < 0.0001) in comparison to control rag2 animals and stayed at constant level up to terminal stage of the disease. MNs count in 4-copy number animal at the age of 150 days did not differ significantly from the control. The number of motor neurons at terminal stage of the disease of 8-copy number animal was considerably lower when compared to rag2 mice (p < 0.0001) and to 4-copy number animal in terminal stage of disease (p < 0.0159) (Fig. 4G). Similarly, total number of neurons (including motor, sensory and inter-neurons) in ventral and dorsal part of spinal cord decreased significantly at 4-copy number mice comparing to rag2 control (p < 0.0001) and remained lower until terminal stage of disease. The number of neurons in 8-copy number mice was visibly lower than rag2 control and 4-copy number mice (Fig. 4H).

**Figure 5:**
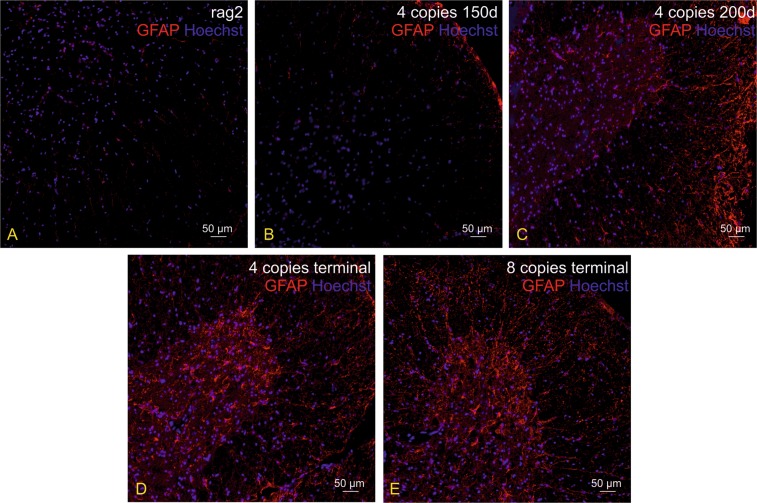
Immunohistochemical analysis illustrating different astrocytes reactivity level (GFAP - red) within the mice spinal cord depending on the age and/or hSOD1 transgene copy number. Nuclei were counterstained with Hoechst 33342 (blue). Astrogliosis was not observed in control Rag2 mice (**A**) and 4-copy number mice at day 150 (**B**). Slight level of astrogliosis was visible in 4-copy number animals at day 200 (**C**). Severe astrogliosis was noticeable in both 4-copy (**D**) and 8-copy (**E**) number animals at terminal stage (250 and 150 days, respectively).

Astrocyte staining revealed correlation between mice age and/or hSOD1 gene copy number, and level of astrogliosis. Both 4-copy and 8-copy number animals in terminal stage (approximately 250 and 150 days, respectively) demonstrated severe astrogliosis in spinal cords as shown in Fig. 5. 4-copy number mice at day 150 were indistinguishable from control Rag2 mice in terms of poor GFAP expression across the spinal cord. 4-copy number mice at day 200 demonstrated intermediate astrocyte reactivity.

**Figure 6:**
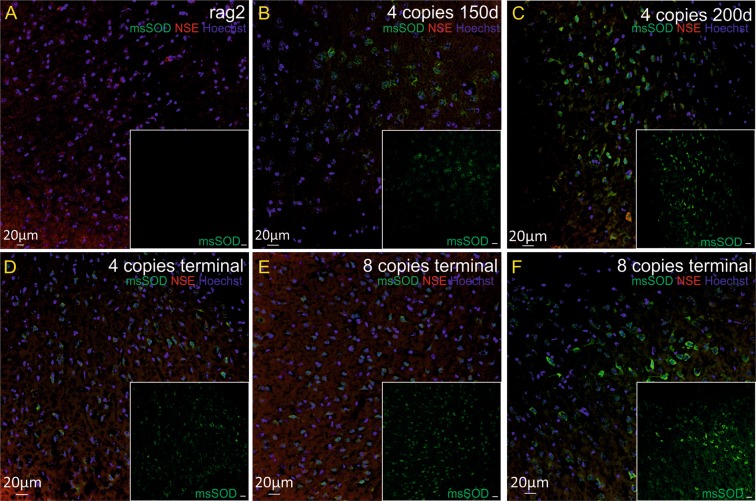
Immunohistochemical analysis illustrating presence of pathological misfolded SOD protein (msSOD – green) in cortical neurons (NSE–red) at all time-points of the disease, independently of the number of hSOD1 copies. Nuclei were counterstained with Hoechst 33342 (blue). (**A**) rag2 control animal, (**B**,**C**) 4-copy SOD1/rag2 mice in pre-symptomatic stage (150 and 200 post-natal day, respectively), (**D**) 4-copy SOD1/rag2 mice in terminal stage of the disease, (**E**,**F**) 8-copy SOD1/rag2 mice in terminal stage of the disease.

**Figure 7:**
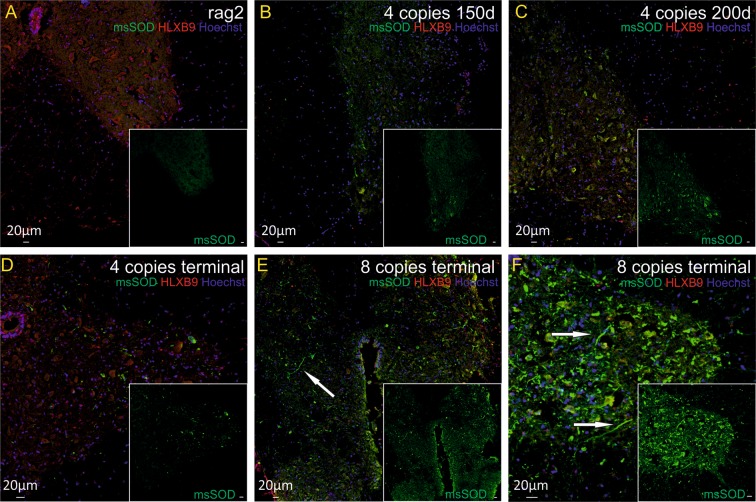
Immunohistochemical analysis illustrating presence of pathological misfolded SOD protein (msSOD–green) in spinal motor neurons (HLXB9 – red) at all time-points of the disease, independently of the number of hSOD1 copies. Nuclei were counterstained with Hoechst 33342 (blue). (**A**) rag2 control animal, (**B**,**C**) 4-copy SOD1/rag2 mice in pre-symptomatic stage (150 and 200 post-natal day, respectively), (**D**) 4-copy SOD1/rag2 mice in terminal stage of the disease, (**E**,**F**) 8-copy SOD1/rag2 mice in terminal stage of the disease.

**Figure 8:**
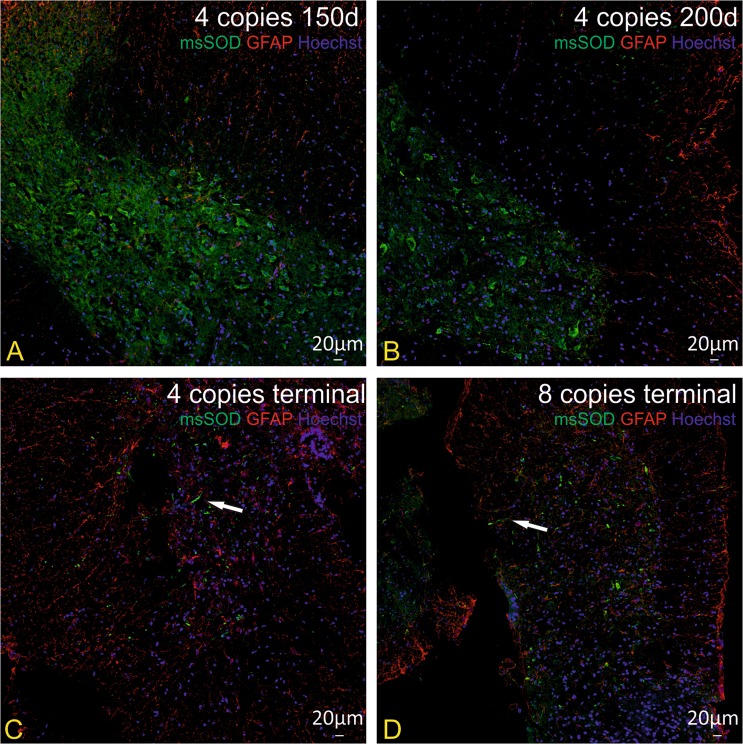
Immunohistochemical analysis illustrating lack of pathological misfolded SOD protein (msSOD–green) in astrocytes (GFAP–red) in spinal cord of SOD1/rag2 mice at all time-points of the disease, independently of the number of hSOD1 copies. Nuclei were counterstained with Hoechst 33342 (blue). (**A**,**B**) 4-copy SOD1/rag2 mice in pre-symptomatic stage (150 and 200 post-natal day, respectively), (**C**) 4-copy SOD1/rag2 mice in terminal stage of the disease, (**D**) 8-copy SOD1/rag2 mice in terminal stage of the disease.

We also wanted to verify if degenerative processes might be due to accumulation of misfolded SOD1 protein in cortical neurons and/or motor neurons or astrocytes in spinal cord of SOD1/rag2 mice. Aggregation of misfolded SOD1 protein was not visible in control brains and spinal cords of rag2 mice. SOD1 aggregates were present in tissues of animals in all investigated time points (Figs 6 and 7). IHC double staining revealed the presence of SOD1 aggregates in cortical neurons of 4-copy SOD1/rag2 mice as early as 150 days postnatally (Fig. 6). Misfolded SOD1 was also present in some of the spinal MNs, especially in the presymptomatic stage of the disease (Fig. 7B,C). However, in terminal stage of the disease localization of msSOD (misfolded SOD) was pronounced in extracellular space of spinal cord. Some of msSOD seemed to localize in axons of spinal neurons (Fig. 7, arrow; Fig. 8, arrow). The accumulation of misfolded protein was restricted to neuronal cells and was not visible in astrocytes (Fig. 8).

### Western blot analysis

We performed western blot analysis to investigate if we would be able to detect the differences in the level of msSOD accumulation in homogenates of cortex and spinal cord of SOD1/rag2 mice. We were able to confirm the results obtained in immunohistochemical analysis. MsSOD was not visible neither in cortex nor in spinal cord of rag2 control mice. We detected msSOD protein in homogenates of cortex and spinal cord of all of the SOD1/rag2 mutants, however we did not see any statistically significant difference in the level of msSOD within the tissues originating from animals at investigated time-points and with either 4 or 8 copy number of SOD transgene (data not shown).

## Discussion

The overall goal of this study was to create relevant ALS model that would be useful in experimental stem cell therapies. Most of the commonly known rodent ALS models are based on fALS mutations such as SOD1, C8OR72, TDP43, FUS, UBQLN2. Some of the mutations are only related to fALS, however in sALS cases mutations like SOD1, TDP43 or UBQLN2 are also present^14^. SOD1 mutation reflects in pathologies i.e. glutamate excitotoxicity, mitochondrial vacuolization, aberrations in axonal transport and astro- and microgliosis leading to MNs degeneration and following muscle weakness, atrophy and eventually death, mostly due to respiratory impairment^15^. All of above-mentioned cellular pathologies are visible in histopathological samples from ALS patients, thus SOD1 mutation-based models seem to be relevant despite some discrepancies, like not pronounced cortical MN degeneration that is a key feature of human ALS pathology. Recently the attention was brought to failed support of glial cells in maintaining motor neuron homeostasis and thus following MNs degeneration in the development of ALS^6,16^. Taking SOD1 mouse into consideration as a potential animal model of ALS, it is worth mentioning that also in case of investigation of glia in ALS pathology, as well as glia replacement therapies, it seems that SOD1 mice are suitable^6,9^.

The choice of the right model in experimental therapies with the use of exogenous cells might be difficult, especially when it comes to investigating cells derived from different species – xenotransplantation. Researchers conducting basic researches who examine various types of cells in rodent models of neurodegenerative diseases struggle with the problem of immunosuppression that not always ensures proper protection of transplanted cells and may eventually affect the final therapeutic effect of the transplants. Therefore, in our studies we decided to combine the ALS mouse model – SOD1 mice and immunocompromised mice with impaired adaptive immune response–rag2 mice. The results of the crossbreeding shed new light not only on the phenotype of mice progeny but also on the crossing paradigm itself. The first important notice was that depending on the females used for breeding (either rag2 or SOD1/rag2, respectively) we obtained mice with symptoms of the disease occurring at different timepoints, which consequently led to variety in lifespan. QPCR-based genotyping revealed differences in the copy number of mutated SOD1 gene across the progeny. We were able to obtain mice with the lowest copy number of SOD1 only when crossing SOD1/rag2 males with rag2 females, suggesting that the progeny had lost some of the copies of transgene. After SOD1/rag2 x SOD1/rag2 crossbreeding mice had either around 4 (3–5) or around 8 (6–9) transgene copies. In compliance with Alexander and co-workers the lifespan of mice bred in our colony was inversely proportional to the number of SOD1 copies^11^. The number of generated 8-copy mice was significantly lower than 4-copy mice (79% of 4-copy comparing to 21% of 8-copy). Decrease in the copy number of SOD1 gene was reported previously during mating process and might be caused by spontaneous intra-locus recombination during meiosis^11,12^. Some of the researchers managed to obtain SOD1 G93A mice with low copy number of the transgene and prolonged survival, however only 80% of mice have shown symptoms of the disease and mice that presented the symptoms had prolonged asymptomatic period and consequently longer mean lifespan in comparison to mice in our colony^12,17^. This fact might be related to different background of our mice, as our colony has mixed background of SOD (B6SJL) and rag2 (129S6) mice. The effect of the genetic background is often omitted, however Heiman-Patterson and collaborators have gathered data proving that disease severity is not only related to the number of SOD1 copies but also to mice origin. This is especially true for congenic lines like NOD or SJL, which demonstrate more severe disease symptoms than those of mixed background B6xSJL^13,18^. It is worth mentioning that this situation might reflect the state in human ALS, especially that of people sharing the same mutation who often demonstrate diverse severity of the disease, implying that there might exists some genetic modifiers that affect disease progression^13^. Impaired immunological response might additionally contribute to severity of the symptoms. Given that in T-cell response during MNs early stage degeneration might activate M2 microglia and slow the loss of motor neurons^19^, lack of adaptive response in SOD/rag2 mice might additionally cause shortened lifespan.

In order to check if we could detect pre-symptomatic changes with the use of non-invasive detection method T2-weighted MR imaging was performed. It appears that during symptomatic period we could easily detect changes in motor nuclei in both 4- and 8-copy mice, however early presymtomatic changes (95 days postnatally) were visible only in case of high SOD copy number mice (8 copies). It is possible that in case of 4-copy SOD mice changes would be visible at later time points (we have used relative dates for both type of mice, the last time point was at 158 postnatal day), however further experiments are needed to explore this area. Evans and co-workers also managed to detect pre-symptomatic changes at 120 postnatal day of SOD1 mice, moreover those changes could be associated with progressing vacuolization, astro- and microgliosis visible in histopathology in the area of motor related nuclei^20^. Another group performed MR imaging in two different ALS mice models based on C57BL6/J or 129S2/SvHsd background, however differences were only visible in mice with C57BL6/J background. The reason for such difference might be explained by different course of pathological events in those two models especially in increased vacuolization in SOD93A C57BL6/J mice^21^. MRI changes in brainstem motor nuclei might reflect degenerative changes in this area including vacuolization, astrogliosis and degeneration of MNs^22,23^. Therefore, it seems that in our model using magnetic resonance imaging might be a reasonable approach to identify neurodegenerative changes non-invasively. It is also important to mention that recent research indicates that muscle atrophy measured by MRI, that is visible already about 56 postnatal day, precedes the visible MRI changes in motor nuclei (noticeable between 70–126 day)^24^. Therefore, for future studies requiring early changes detection, muscle girth measurements would be worth considering.

Next, we investigated the presence and condition of motor neurons in spinal cord of SOD1/rag2 mice at different time points. Some degenerating neurons were visible already at 150 postnatal day in 4-copy mice, however degeneration was not obvious across all animals. Some of the 200 days-old 4-copy mice presented MNs that were smaller and/or degenerating. However, IHC analysis did not reveal dramatic changes also at that time point. It is likely that degeneration of MNs in spinal cord is not pronounced yet to be visualized via immunohistochemical staining, however changes like cytoplasmic vacuoles and mitochondrial pathologies might already be visible via electron microscopy. In classic SOD1 G93A mice model, which lives around 130 days, first pathological changes of MNs like vacuolization are visible as early as 75 days postnatally. At the same time-point changes in MN count were reduced by 20–30% comparing to control mice^25,26^. In our model we were able to observe decrease in the number of motor neurons as early as 200 days in 4 copy number mice and remained low until terminal stage of disease. Similarly to classic SOD1 G93 model, the MN number was 20% lower in comparison to control mice^25,26^. Thus, our experiments revealed that in 4 copy number mice degenerative changes of MN appear around the age of 200 days. Therefore, when optimizing the time window for treatment it might be crucial to supply neuroprotective agents before degeneration of MNs occur. Despite not pronounced changes in MNs deterioration at the age of 150 days, all of the terminal stage animals, whether 4- or 8-copies, displayed diminished number of MNs and reduced size of the cells. Previous studies have shown astrocytes activation in post-mortem tissue of ALS patients and spinal cord samples of human msSOD1-expressing mutant mice. This implies that astrogliosis may have a role in ALS pathogenesis^27^. Therefore, we decided to verify if we could see any alterations in astrocytes of spinal cord slices. Our findings are consistent with previous studies and confirm severe astrogliosis in SOD1 mutant mice at terminal stage regardless of transgene copy number.

Recent reports underline the role of misfolded SOD1 protein in pathology of familiar ALS. The SOD pathology is mostly related to gain-of-function of superoxide dismutase 1 that leads to aggregation tendency. What is more, mutated protein has inclination to seed misfolding to wild type protein and furthermore spread across the neighbouring cells^28^. In our model already at the earliest investigated time point - 150 post-natal day, we observed pathology associated with aggregation of misfolded SOD1 protein that was not visible in rag2 control mice. Aggregation of msSOD was related to neuronal cells and was visible across all investigated time points. What is more, in the terminal stage of the disease localization pattern of misfolded SOD1 in spinal cord has changed. MsSOD1 was visible also in extracellular space of grey matter of spinal cord. Such result might be due to MN degeneration and aggregates released outside of the cell body.

## Conclusions

We managed to obtain a novel model of ALS: a stable line of SOD1/rag2 immunocompromised mouse. Despite various number of mutated SOD1 transgene across the mice population, through genotyping we were able to predict disease severity. Our new model demonstrates pathologies typical for classic G93A SOD1 mice model: changes in MNs and toxic aggregates of misfolded SOD1 protein. Finally, we are adamant that in the future similar MRI-based studies might be useful in non-invasive analysis of ALS disease progression.

## Materials and Methods

All experiments were performed in accordance with the guidelines and regulations of I Local Ethics Committee for Animal Experiments in Warsaw and were approved by the 37/2017 and 240/2017 acts. We crossed animals in two different breeding patterns. Phenotypes of offspring varied between groups in terms of lifespan and symptoms onset. To establish the genotype of double mutants we performed real-time PCR.

### Genotyping

#### Isolation of genomic DNA

Genomic DNA was isolated from ear pinna of 3-week old mice. The procedure was performed using High Pure PCR template preparation kit (Roche). Briefly, tissue was digested with proteinase K and lysis buffer in 55 °C and constant vortexing for 12 h. The following steps of isolation were handled as described in the manufacturer’s protocol. DNA was separated on the mini-columns and after the final eluting step the amount of DNA was measured using Nanodrop (Thermo Fisher Scientific).

#### PCR

The standard PCR was performed using primers for hSOD1 transgene and IL2 as an internal control. Primers sequences as well as PCR conditions were established by the manufacturer (Jackson Laboratory) and are presented in Table 1. The following program was set: initial denaturation: 95 °C, 3 min.; denaturation: 95 °C, 30 sec.; primers annealing: 61 °C, 30 sec.; elongation: 72 °C, 45 sec.; final elongation: 72 °C, 2 min. Steps were repeated for 35 cycles.

**Table 1:** PCR primers sequence.

Primer	Sequence 5′- >3′
hSOD1 forward	CAT CAG CCC TAA TCC ATC TGA
hSOD1 reverse	CGC GAC TAA CAA TCA AAG TGA
IL2 forward	CTA GGC CAC AGA ATT GAA AGA TCT
IL2 reverse	GTA GGT GGA AAT TCT AGC ATC ATC C

To prepare PCR reaction we used PCR buffer 10× (Applied Biosystems), buffer Q 5× (Applied Biosystems), Taq polimerase 1U (Applied Biosystems), dNTPs 2.0 mM (Applied Biosystems), 20 pmol of primers. The PCR products were placed in 1.5% agarose gel and electrophoresis was performed at 100 mA for 1 h. The homozygous SOD1 mice are lethal therefore, for further experiments hemizygous animals were selected based on presence of mutated SOD1 gene.

#### Real-time PCR

It was recently proved that lifespan and progression of the disease in ALS mouse models depends on the copy number of mutated SOD1 gene. Because we have noticed different disease manifestation time across mice in our colony we performed quantitative PCR in order to evaluate the exact copy number of mutated SOD1 gene. We used SOD1 mouse from Jackson Laboratory as a control animal with 10 copies of SOD1 transgene. 50 ng of genomic DNA was used as a template for real time PCR. TaqMan probes and primers (Thermo Fisher Scientific) used for SOD1 gene are presented in Table 2, ApoB was used as a reference (Table 2). Probes and primers sequences were based on Jackson Laboratory’s protocol (available on the web page). Probes were associated with either FAM or VIC fluorescent dyes and MGB as a quencher. We used standard program for real-time PCR that included following steps: holding stage: 50 °C, 2 min.; cycling stage: 40× (95 °C, 1 sec.; 60 °C, 20 sec.). The results were analysed through relative analysis - comparing the ΔΔCt (ΔCt Apo – ΔCt SOD) of mice from our colony to ΔΔCt of a control SOD1 mice. The copy number of SOD1 transgene was calculated according to the following equation: Copy Number = 10 × 2^(ΔΔCt probe − ΔΔCt ctrl)^.

**Table 2:** Real-time PCR primers and probes sequence.

Primer/Probe	Sequence 5′- >3′
hSOD1 forward	GGG AAG CTG TTG TCC CAA G
hSOD1 reverse	CAA GGG GAG GTA AAA GAG AGC
ApoB forward	CAC GTC GGC TCC AGC ATT
ApoB reverse	TCA CCA GTC ATT TCT GCC TTT G
hSOD1 probe	CTG CAT CTG GTT CTT GCA AAA CAC CA
ApoB probe	CCA ATG GTC GGG CAC TGC TCA A

### Magnetic Resonance Imaging (MRI)

MR imaging was performed using 7T MR tomograph (BioSpec 70/30 USR, Bruker, Ettlingen, Germany). SOD1/rag2 mice with different copy number were scanned at the final stage of the disease in order to verify if there are any structural changes within the brain. The animals (n = 5 per group) having either low or high copy number of hSOD1 transgene were submitted to MR scanning monthly at the age of 3, 4 and 5 months to screen for any anatomical differences in brain anatomy. For MR imaging mice were anesthetized with 1.5–2% isofluorane in oxygen and positioned head-first prone in the MR compatible animal bed. A transmit cylindrical radiofrequency coil (8.6 cm inner diameter, Bruker) and a mouse brain dedicated receive-only array surface coil (2 × 2 elements, Bruker) were positioned over the animal’s head. Respiration rate and body temperature were monitored throughout the experiment with a small animal monitoring system (SA Instruments, Stony Brook, NY, USA). Positioning tri-pilot scans were performed, followed by high resolution T_2_-weighted TurboRARE structural scan (T_R_/T_Eeff_ = 7000/15 ms, RARE factor = 4, spatial resolution = 86 × 86 × 350 μm, FOV = 22 × 22 mm, 42 slices, no gap, NEX = 4, Scan Time = 23 min) covering the whole brain.

### Immunohistochemical analysis (IHC)

#### Tissue preparation

After genotyping and confirming the exact number of copies of transgene and observation of lifespan of animals in our colony, mice were divided into 3 separate groups (based on the predicted time of survival). Animals with very low copy number – 0-3 copies (i), low copy number 4–5 copies (ii) and high copy number – 6–9 copies (iii) of transgene differed in survival time and symptoms onset. Therefore, in order to observe the differences in morphology between groups we divided them into 4 subgroups: low-copy 150 days, low copy 200 days, low copy terminal (around 250 days), high copy terminal (around 150 days). The animals were sacrificed and perfused with 4% paraformaldehyde (PFA; Sigma). The brains and spinal cords were extracted and frozen in −80 °C. Tissues were sectioned into 25 µm slices on cryostat and frozen in −80 °C for further analysis.

#### Immunohistochemistry

IHC was performed to validate the number and state of motor-neurons in SOD/rag2 mice with different number of copies of SOD1 transgene and verify if misfolding of SOD1 protein occurs in investigated mouse model. In order to block unspecific antibody binding sites slices were incubated in blocking solution (5% BSA, 10% NGS, 0.25% Triton X-100 in PBS) for 1 h in room temperature (RT). Next, tissues were treated (overnight in 4 °C) with primary antibodies: rabbit anti-HLXB9 (1:200, Thermo Fisher Scientific) for motor neurons and mouse anti-human misfolded SOD1 clone B8H10 (1:200, MediMabs) – for detection of misfolded SOD1 protein. To identify mature neurons, we used mouse anti-NSE (neuron specific enolase) antibody (1:200, Chemicon). In order to detect astrocytes, we used rabbit anti-GFAP antibody (1:500, Dako). Following three washing steps with PBS tissues were incubated for 1 h in RT with appropriate secondary antibodies: goat anti- rabbit Alexa Fluor 546 (1:500, Thermo Fisher Scientific) and goat anti-mouse IgG1 Alexa Fluor 488 (1:500, Thermo Fisher Scientific) or and goat anti-mouse IgG2a Alexa Fluor 546 (1:500, Thermo Fisher Scientific). After washing procedure slices were incubated for 20 min in RT with Hoechst 33342 (200 µg/ml, Sigma) for nuclei counterstaining. Following PBS washing slides were closed with coverslips using fluorescent mounting medium (Dako). Visual analysis was performed with Carl Zeiss Confocal microscope LSM 780 and Carl Zeiss Cell Observer where the pictures of the whole spinal cord cross sections were captured using tile-scan command.

In order to preform motor neuron counting we used Nissl staining. Briefly, slices were washed with distilled water, incubated with 0.1% cresyl violet solution for 15 min and then washed again with distilled water. Next, samples were washed in turn with 70%, 96%, 100% ethanol, and finally in xylene. Finally, sections were immersed with differentiation solution (2 drops of glacial acetic acid in 95% ethanol), washed two times in 100% ethanol, cleared in xylene and mounted with DPX mounting medium under a coverslip.

### Western Blot analysis

Mice tissues were homogenized and lysed in RIPA buffer. Samples containing 10 μg of total protein fraction were separated by SDS-PAGE and transferred to PVDF-membranes. Membranes were then incubated in 5% non-fat dried milk dissolved in Tris-buffered saline containing Tween-20 (TBST) for 1 hour in room temperature (RT), and incubated with primary antibodies at 4 °C overnight. The next day, membranes were washed three times in TBST and incubated with secondary antibody for 1 hour in RT. Membranes were then again washed three times in TBST and incubated for 5 min in Amersham ECL Western Blotting Detection Reagent (GE Healthcare) according to manufacturer’s protocol. Proteins were visualized using G:BOX and GeneSys software (Syngene). The following antibodies were used: primary: 1:250 mouse anti-misfolded human SOD1, clone B8H10 (MediMabs) and 1:500 mouse anti-actin, clone C4 (MP Biomedicals); secondary: 1:4000 goat anti-mouse IgG, Fab-specific (Sigma). The band density was normalized to actin.

### Statistical analysis

In order to analyse the survival of SOD1/rag2 mice depending on the copy number of transgene we used Kaplan-Meier estimate (number of mice used in the study: for 0–3 transgene copies: n = 36 mice; for 4–5 copies: n = 36 mice; for 6–9 copies: n = 32 mice). To verify if there is a difference in lifespan between groups we used log-rank test (Mantel-Cox and log rank test for trend). Correlation coefficient – r, and p-value (two-tailed) was calculated to estimate the relationship between the number of SOD1 copies within the animal genome and its lifespan. We performed linear regression analysis to check if we were able to predict the animal lifespan on the basis of SOD1 gene copy number. All statistical analysis was conducted using Prism 7.04 software (GraphPad). To verify dependency between the number of motor-neurons, the age of an animal and the SOD1 copy number we performed One-Way ANOVA test with Brown-Forsythe post-hoc test and Tukey’s multiple comparison test. In this study we counted neurons from ventral and dorsal horn of spinal cord from at least 5 sections separated from each other by 75–100 µm (n = 4 for rag2 animals, high copy, terminal group; n = 5 in case of low copy animals).

## Data Availability

The authors confirm that all data underlying the findings are fully available from the corresponding author on reasonable request.
